# The H3K27 demethylase controls the lateral line embryogenesis of zebrafish

**DOI:** 10.1007/s10565-021-09669-y

**Published:** 2021-10-29

**Authors:** Dongmei Tang, Yitong Lu, Na Zuo, Renchun Yan, Cheng Wu, Lijuan Wu, Shaofeng Liu, Yingzi He

**Affiliations:** 1 ENT Institute and Department of Otorhinolaryngology, Eye & ENT Hospital, State Key Laboratory of Medical Neurobiology and MOE Frontiers Center for Brain Science, NHC Key Laboratory of Hearing Medicine, Fudan University, 83 Fenyang Road, Shanghai, 200031 China; 2 Department of Otolaryngology-Head and Neck Surgery, Yijishan Hospital of Wannan Medical College, 2 Zheshanwest Road, Wuhu, 241001 Anhui China

**Keywords:** *kdm6bb*, Primordium migration, Cell proliferation, Chemokines, Fgf signaling

## Abstract

**Background:**

Kdm6b, a specific histone 3 lysine 27 (H3K27) demethylase, has been reported to be implicated in a variety of developmental processes including cell differentiation and cell fate determination and multiple organogenesis. Here, we regulated the transcript level of *kdm6bb* to study the potential role in controlling the hearing organ development of zebrafish.

**Methods:**

A morpholino antisense oligonucleotide (MO) strategy was used to induce Kdm6b deficiency; immunohistochemical staining and in situ hybridization analysis were conducted to figure out the morphologic alterations and embryonic mechanisms.

**Results:**

*Kdm6bb* is expressed in the primordium and neuromasts at the early stage of zebrafish embryogenesis, suggesting a potential function of Kdm6b in the development of mechanosensory organs. Knockdown of *kdm6bb* severely influences the cell migration and proliferation in posterior lateral line primordium, abates the number of neuromasts along the trunk, and mRNA-mediated rescue test can partially renew the neuromasts. Loss of *kdm6bb* might be related to aberrant expressions of chemokine genes encompassing *cxcl12a* and *cxcr4b/cxcr7b* in the migrating primordium. Moreover, inhibition of *kdm6bb* reduces the expression of genes in Fgf signaling pathway, while it increases the *axin2* and *lef1* expression level of Wnt/β-catenin signaling during the migrating stage.

**Conclusions:**

Collectively, our results revealed that Kdm6b plays an essential role in guiding the migration of primordium and in regulating the deposition of zebrafish neuromasts by mediating the gene expression of chemokines and Wnt and Fgf signaling pathway. Since histone methylation and demethylation are reversible, targeting Kdm6b may present as a novel therapeutic regimen for hearing disorders.

**Supplementary Information:**

The online version contains supplementary material available at 10.1007/s10565-021-09669-y.

## Background

The malformation of inner ear structure during the development process is a leading cause of congenital hearing loss. Multiple regulatory factors are involved in the developmental event of otic morphogenesis, but the underlying molecular mechanisms have not yet been entirely revealed. The lateral line (LL) system in zebrafish, an organ to sense water pressure and movements (Engelmann et al. 2000; Ghysen and Dambly-Chaudiere 2007; Ma and Raible 2009), is similar in structure and function with the vertebrate inner ear (Nicolson 2005; Whitfield 2002), which has been proven to be an excellent model to understand the fundamental principles in the development of sensory organs. As the mechanosensory organs of lateral line, neuromasts are divided into the cephalic anterior LL and the posterior LL which are distributed along the whole body of zebrafish (Aman and Piotrowski 2009; Dalle Nogare et al. 2014; Gallardo et al. 2010). Directional cell cluster migration and neuromast deposition are the fundamental behavior at the early stage of the development period of posterior lateral line (PLL), that the migration begins around 22 h post-fertilization (hpf) and quits at approximately 42 hpf in zebrafish, while the deposition starts once the fourth rosette of primordium is assembled, and ends in five to six trunk neuromasts and two or three terminal neuromasts between 25 and 54 hpf (Nechiporuk and Raible 2008). The microstructure of the migrating PLL is a series of rose-shaped organs, called rosettes, which deposits at regular intervals (Dambly-Chaudiere et al. 2003; Ghysen and Dambly-Chaudiere 2004). Mature neuromast consists of central hair cells (HCs) and surrounding supporting cells (SCs) as well as the periphery mantle cells. Previous studies have unmasked refined coordination involving the canonical Wnt pathway, Fgf family, Notch signaling, and the chemokine pathway during the posterior LL cluster cell migration and neuromast morphogenesis, which are the basics of normal mechanosensory behaviors (Aman and Piotrowski 2008; Lecaudey et al. 2008; Nechiporuk and Raible 2008). However, the dynamic and modifiable regulation tools in the development process are rarely reported.

The epigenetic mechanisms, characterized in changing gene expression but not DNA sequence, are indispensable to multiple development events. DNA methylation and histone modifications, including histone acetylation and histone demethylation, are the most important covalent modification forms catalyzed by a series of enzymes. By alterations of histone proteins at specific residues, lysine demethylases (KDMs) and lysine methyltransferases (KMTs) are widely involved in regulating gene expression during the developmental process (Clarke et al. 2020). The H3K27 demethylase Kdm6b (also known as Jmjd3) is considered to be an H3K27 demethylase that can specifically mediate demethylation of H3K27me2/3 peptides (Agger et al. 2007; Hong et al. 2007; Xiang et al. 2007; Yang et al. 2019). Through dynamic methylation and demethylation, epigenetic modification plays important roles in regulating many cellular processes; for example, trimethylated H3K27 is generally reported as a mark for gene repression, while monomethylated H3K27 is related to gene activation (Hong et al. 2007; Xiang et al. 2007). Recently, Kdm6b has been proposed to be involved in controlling differentiation of embryonic stem cells, endochondral and osteogenic organogenesis, and lung formation (Akerberg et al. 2017; Li et al. 2014; Ye et al. 2012; Zhang et al. 2015). In other species outside vertebrates, for example, oyster and mangrove rivulus, histone methylation was closely involved in their early development process (Fellous et al. 2019, 2015). Additionally, Kdm6b deficiency through knockdown or knockout strategy can inhibit the cell growth and survival of multiple myeloma cells, suggesting a central role in tumorigenesis (Ohguchi et al. 2017). Kdm6b has recently been described as a regulator in temperature-dependent sex determination by activating the male pathway (Ge et al. 2018; Weber et al. 2020). As reported, *Jmjd3* functionality lost resulted in an inability to differentiate bipolar cell subpopulation, implying that the Jmjd3 is critical for the development and maturation of retinal cells (Iida et al. 2014). Taken together, growing evidence shows that Kdm6b is associated with many human diseases, for example, developmental diseases, cancer, immune systematic diseases, infection, and urinary systems disorders (Zhang et al. 2019). However, it remains unknown whether Kdm6b plays any role in the development of hearing organs.

In this study, we identified *kdm6bb* expression in the developing sensory organs of zebrafish. By specific morpholino targeting Kdm6b, we demonstrated that interference with Kdm6b expression resulted in a disarranged PLL pattern, destruction of cell proliferation, and a reduced number of neuromasts after deposition period in dose-dependent effect. In addition, the in situ hybridization analysis showed that Kdm6b regulated the posterior zebrafish lateral line by the chemokines and Fgf pathways and that the *cxcl12a/cxcr4b/cxcr7b* ligand-receptor system is significantly inhibited; the expression of Fgf family members *fgf3*, *fgf10*, *pea3*, and *fgfr1* are all decreased; while the Wnt/β-catenin targets *axin2* and *lef1* restricted in the leading zone highly expressed into an expansion range. Our study uncovered that Kdm6b is essential for the development of the posterior LL system of zebrafish and its reversible modification might be a useful target in improving hearing level.

## Results

### Detection of Kdm6b in the developing sensory organs of zebrafish

**Fig. 1:**
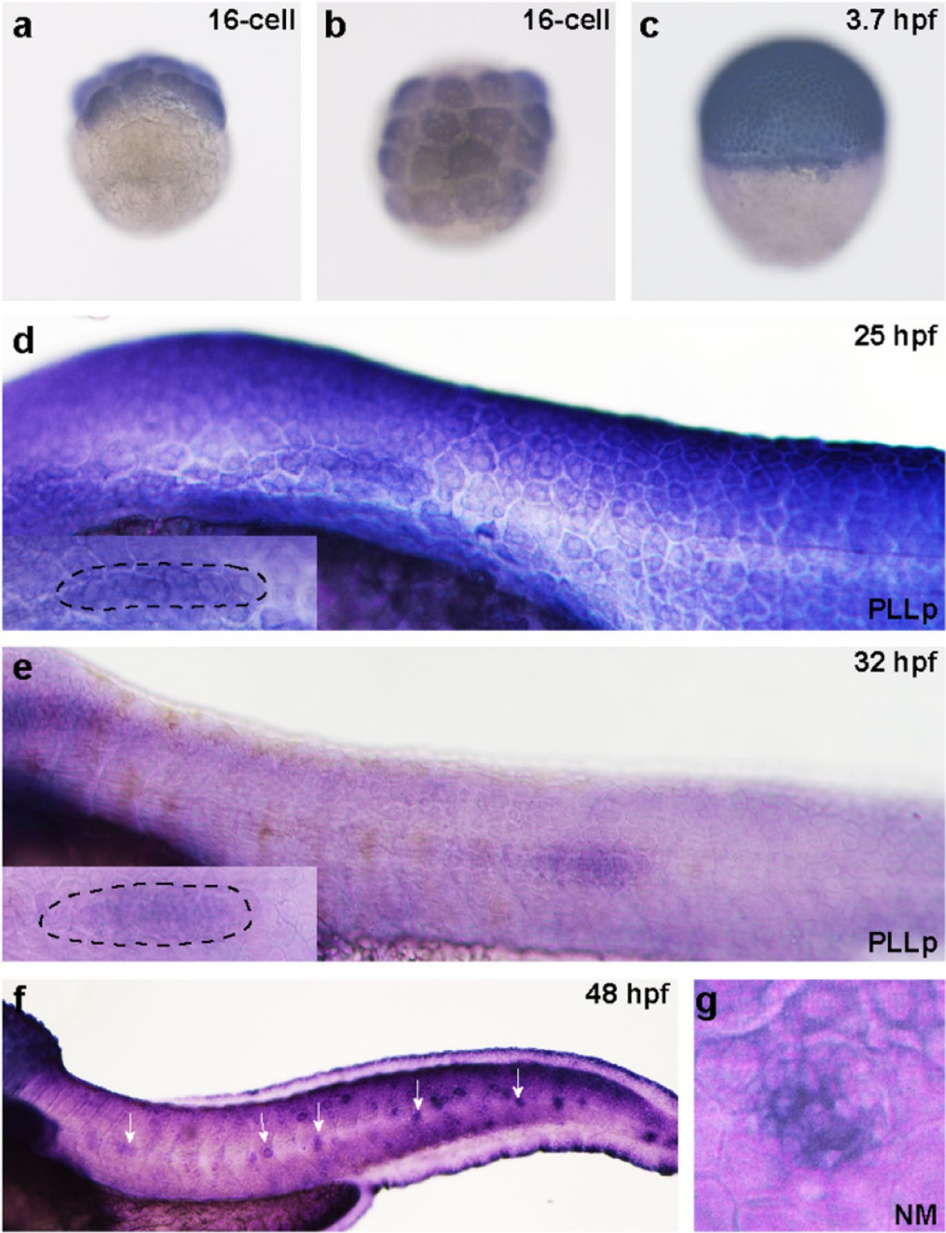
The expression pattern of *kdm6bb* during the early development stage of zebrafish. **a, b** *Kdm6bb* is detected at the early embryonic stage at 16-cell from the lateral and top view (**a**, **b**; *n* = 14 embryos) and blastula period (3.7 hpf) (**c**; *n* = 12 embryos) by whole-mount in situ hybridization. **d–f** *Kdm6bb* is expressed in the migrating primordium (**d**, **e**) and deposited neuromasts (**f**). The dotted lines of (**d**; *n* = 8 embryos) and (**e**; *n* = 10 embryos) outline the migrating primordia at higher magnification. White arrows in (**f;** *n* = 16 embryos) mark the neuromasts stained with *kdm6bb* along the trunk. The details of *kdm6bb* staining in magnified neuromasts are presented in (**g**). The experiment was duplicated for two times

We first used WISH analysis to detect whether Kdm6b is expressed in the developing zebrafish embryo and to figure out its expression pattern. We collected the embryo in chronological order from 16-cell stage to 48 hpf according to the stage series by Kimmel CB et al. (Kimmel et al. 1995). We conducted both the sense and antisense mRNA probe for *kdm6bb*. However, we failed to detect any expression of Kdm6b in the whole body of zebrafish, and the representative AS and S pictures were shown in Figure S1. We found Kdm6b was expressed in the blastomeres at 16-cell stage from lateral and top view (Fig. 1a–1b). At 3.7 hpf, Kdm6b was detected in the elliptical shape (Fig. 1c). At 25–32 hpf, Kdm6b was found prominently expressed in the migrating primordium (Fig. 1d–1f). By 48 hpf, Kdm6b staining was found in the deposited posterior lateral line (PLL) neuromasts and mainly focused on the central hair cell region (Figs. 1f and 1g).

### Kdm6b is required for the normal depositing pattern in the lateral line system

Since the whole-mount ISH data showed evident expression of Kdm6b in the PLL neuromasts, we studied whether Kdm6b is required during neuromasts deposition. We chose the transgenic line *Tg (cldnb: lynGFP)* zebrafish as experimental models for more direct observation on the primordium migration and neuromast deposition since green fluorescent protein (GFP) is expressed in the cell membrane of primordium and neuromast.

**Fig. 2:**
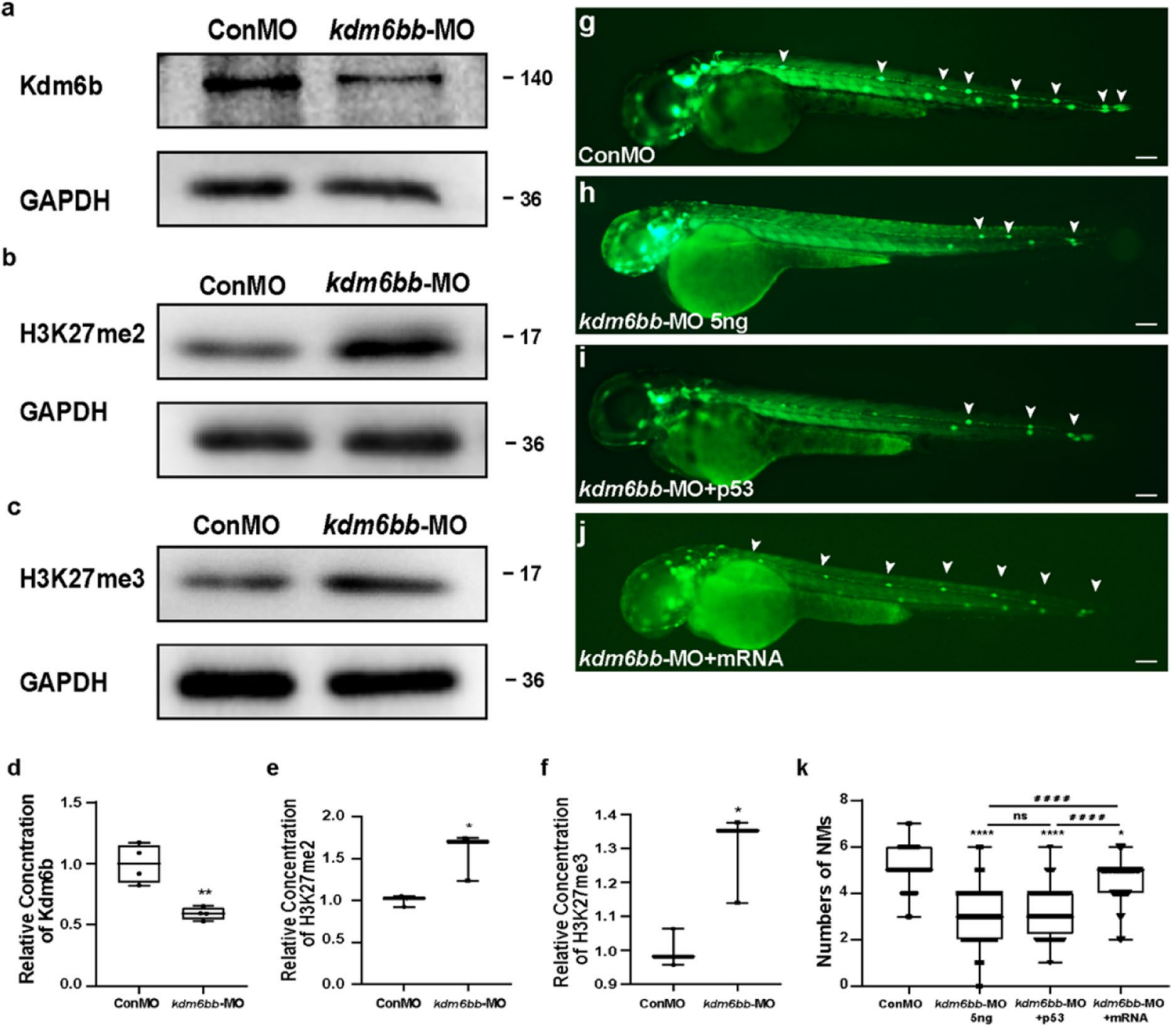
Kdm6b is required for cell migration and neuromast deposition in zebrafish posterior lateral lines. **a–f** Kdm6b level is effectively downregulated using the morpholino technology. The protein blotting of Kdm6b is significantly decreased (**a**), and the protein blottings of H3K27me2 (**b**) and H3k27me3 (**c**) were both upregulated by the special antisense morpholino injection both in the band intensity. **d–f** The quantification analysis of relative concentration of Kdm6b, H3K27me2, and H3K27me3. Data are recorded as mean (minimum and maximum values). *p < 0.05 and **p < 0.01. **g–j** At 48 hpf, the deposited neuromasts are labeled in green along the posterior body of control embryos (**g**), Kdm6b-deficient mutants (**h**), co-injection of *kdm6bb*-MO + p53 (**i**), and co-injection of *kdm6bb*-MO + *kdm6bb* mRNA specimens (**j**). The neuromasts of PLL are labeled in green fluorescence in the transgenic *cldnb:lynGFP* embryos, and a severe reduction in number of neuromasts is found (**g–h**). The decreased number of neuromasts was confirmed when co-injected with p53 and *kdm6bb*-MO (**i**). The decreased number of neuromasts by Kdm6b-defect is partially rescued by the combined injection of *kdm6bb* mRNA (**j**). White arrowheads label the neuromast along the trunk and terminal of the posterior LL (**g–j**). **k** Statistical analysis of the number of posterior LL neuromasts at 48 hpf in controls (*n* = 185), Kdm6b-deficient embryos (*n* = 129), and *kdm6bb*-MO + mRNA members (*n* = 42). ****p < 0.0001 (the contrast between *kdm6bb*-MO group with Con-MO group), ^####^*p* < 0.0001 (the contrast between *kdm6bb*-MO group with *kdm6bb*-MO + mRNA group), *p < 0.05 (the contrast between *kdm6bb*-MO + mRNA group with Con-MO group). Scale bars mark a distance of 100 μm

To investigate whether Kdm6b has role on the development of zebrafish, we first introduced a translational antisense morpholino (MO) strategy to downregulate the Kdm6b level. To exclude the effect of injection operation, the control group was injected with control-MO. We used Western blotting analysis to detect the efficacy of knockdown strategy and found a significant reduction of Kdm6b level after injection with *kdm6bb*-MO compared to that injected with Con-MO (Fig. 2a and 2d). Besides, we also detected the protein levels of H3K27me2 and H3K27me3 and confirmed that H3K27me2/3 were both upregulated when the level of Kdm6b was knockdown by *kdm6bb*-MO (Figs. 2b-2c, and 2e-2f).

At 48 hpf, a stage when PLL primordium stops to migrate and finishes deposition, an average of 5.065 ± 0.056 neuromasts (*n* = 185) was distributed periodically over the trunk in the embryos injected with control-MO (Fig. 2g and 2k). In contrast, the average number of neuromasts was significantly reduced to 3.116 ± 0.091 (*n* = 129) after injection with *kdm6bb*-MO (Fig. 2h and 2k). Interestingly, we did not examine any significant difference in the number of terminal neuromasts between the experiment and the control group. According to the previous study, up to 18% of morpholinos are in the possibility of off-target, resulting in cell death or central nervous system (CNS) destruction mainly on the cause of *p53*-mediated apoptosis (Ekker and Larson 2001; Robu et al. 2007). To avoid the non-specific effect of morpholinos, we co-injected *p53* with *kdm6bb* morpholinos and found a severe reduction in the number of neuromasts, similar to those injected with KDM6B-MO only, confirming a powerful efficacy of *kdm6bb*-MO (Fig. 2h and 2i).

To further validate the role of Kdm6b in embryonic PLL morphogenesis, *kdm6bb* mRNA and *kdm6bb*-MO were injected together. Co-injection of *kdm6bb* mRNA and morpholino could partially rescue the defects of posterior LL, and the reduced number of neuromasts on the body trunk was restored to 4.667 ± 0.111 (*n* = 42) (Fig. 2j and 2k). Taken together, these conclusions can be illustrated that the deletion of *kdm6bb* affects the normal PLL morphogenesis during the development of zebrafish embryos.

### Kdm6b knockdown disrupts normal rosette assembly and cell proliferation of the primordium

**Fig. 3:**
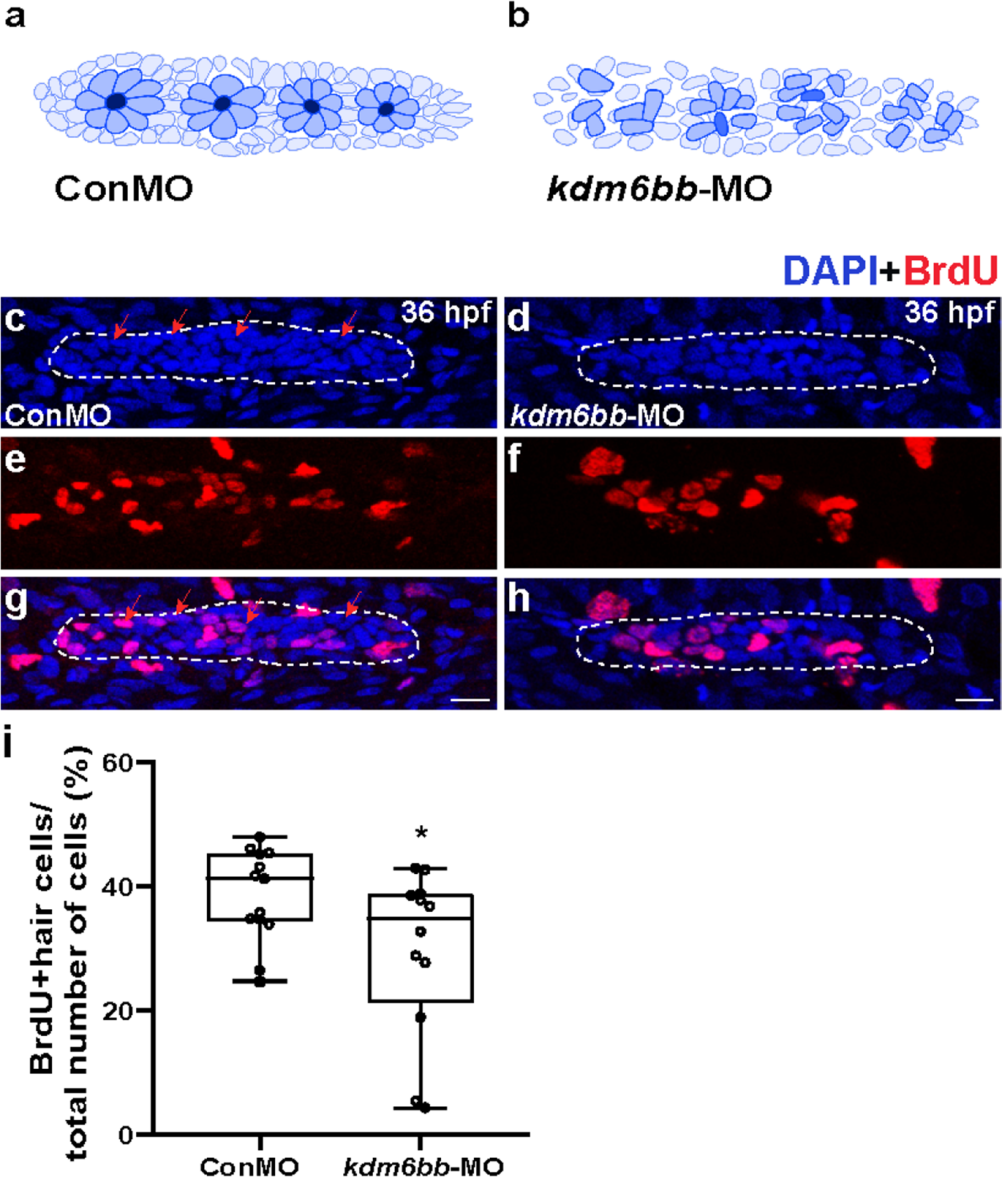
The downregulation of Kdm6b represses cell proliferation and disrupts rosette assembly during primordium migration. **a, b** The diagram of rosette organization in controls and *kdm6bb*-MO embryos. **c–h** Representative images show the comparison in number of BrdU labeled proliferating cells between controls embryos (**c, e, g**) and Kdm6b-deficient mutants (**d, f, h**) at 36 hpf. Red arrows indicate the rosette-shaped clusters of the primordium (**c**). Scale bars mark the 10 μm scale. **i** Significant difference in quantification of BrdU index in control embryos and *kdm6bb*-MO embryos. Data are recorded as mean (minimum and maximum values). *p < 0.05

At the early stage of lateral line development, the primordium is migrating forward to the tail region and is organized into rosettes in the trailing zone. Neuromast deposition occurs when the fourth rosette is assembled. However, in the *kdm6bb* morphants, we did not find a normal rosette pattern at 36 hpf when compared with the corresponding control primordia, suggesting obvious damage of rosette assembly by inhibition of *kdm6bb* (Figs. 3a-3d).

Cell proliferation has been reported to be widely involved in many processes such as the size of primordium, the capacity of migration, and the deposition cycle of proneuromasts in the migrating primordium (Aman et al. 2011). In our previous study, we found GSK-J4 (a selective inhibitor of H3K27me3 histone demethylase) treatment prevented both hair cell and supporting cell regeneration through reducing proliferation (Bao et al. 2017). Here, we investigated whether cell proliferation behavior is vitiated in Kdm6b-deficient mutants during the zebrafish embryo development. BrdU staining was used to capture the proliferating cells in the primordia of *kdm6bb* mutants and control embryos at 36 hpf. The BrdU index (the ratio of BrdU-marked nuclei to total cell nuclei) was significantly decreased in the *kdm6bb*-MO primordia compared to the parallel controls, while the BrdU index was 29.56% ± 0.04 (*n* = 13) in *kdm6bb* mutants and 38.45% ± 0.02 (*n* = 12) in control siblings, respectively (Fig. 3e–3i). Collectively, our data manifested that Kdm6b knockdown induces inhibited cell proliferation and apoptosis during PLL development, resulting an abnormal assembly of classical rosettes.

### Kdm6b downregulation impacts primordium migration by restriction of chemokine signaling

**Fig. 4:**
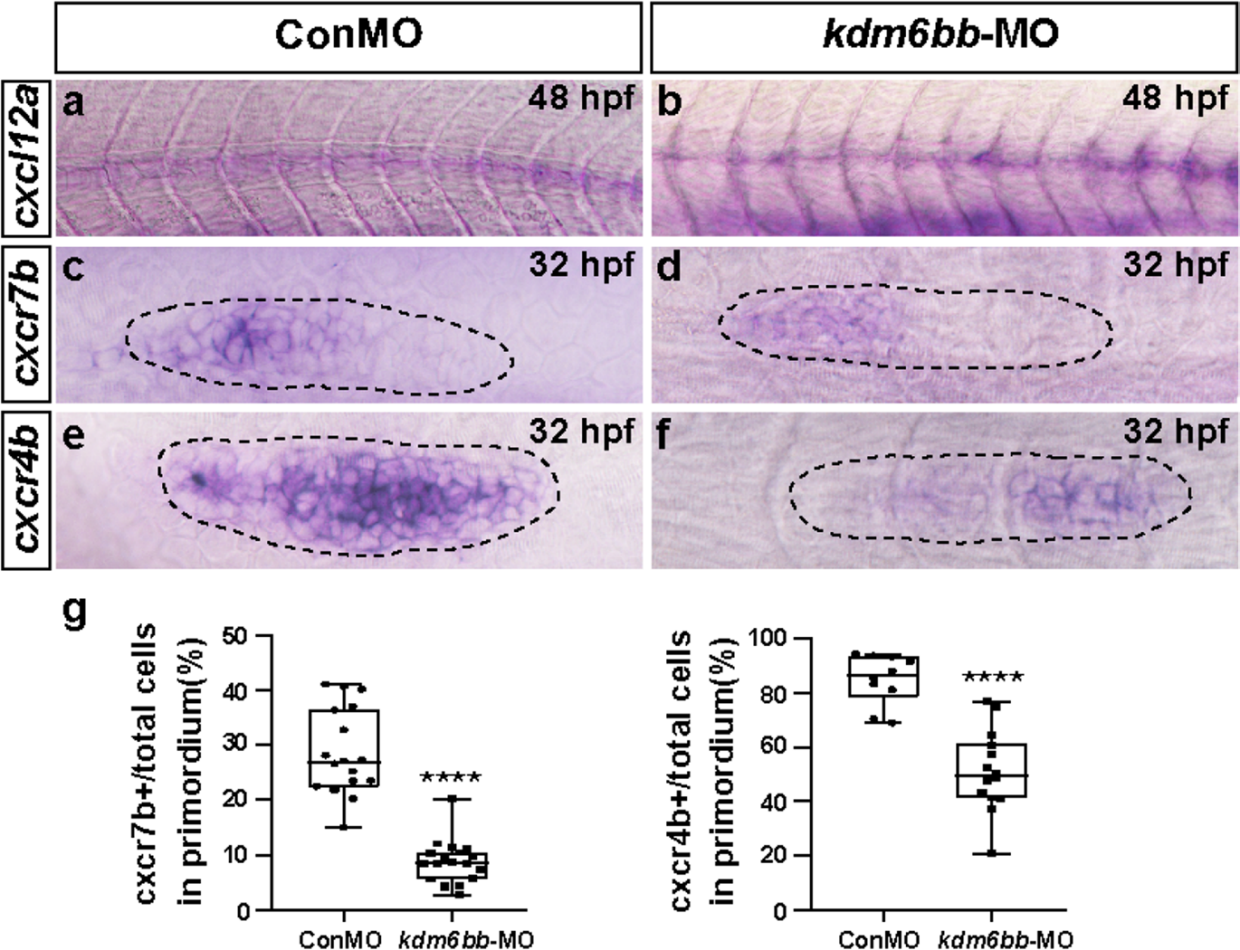
The chemokine signaling pathway is disrupted by *kdm6bb*-MO. **a–f** Representative in situ staining of chemokine members *cxcl12a*, *cxcr4b*, and *cxcr7b* are compared between the controls and *kdm6bb* morphants at 32–48 hpf. (**a**, **b**)The consecutive expression of *cxcl12a* is interrupted in Kdm6b knockdown embryos, both (**c**, **d**) *cxcr4b* and (**e**, **f**) *cxcr7b* expressions are downregulated in *kdm6bb* morpholino mutants in comparison with the control-MO-injected embryos. **g** The ratio of *cxcr7b* and *cxcr4b*-positive cells to total cells in the primordia of controls (ConMO; *n* = 18 embryos for *cxcr7b*, and *n* = 10 embryos for *cxcr4b*) and experiment group (*kdm6bb*-MO; *n* = 19 embryos for *cxcr7b*, and *n* = 14 embryos for *cxcr4b*). The black dotted lines outline the primordium appearance. The leading region is positioned to the right and the trailing region is to the left. Data are recorded as mean (minimum and maximum values). ****p < 0.0001

Chemokines and coupled receptors are active in promoting cell proliferation and in guiding the direction of migrating cells (Wang and Knaut 2014). Of the detected chemokine ligands and receptors in humans and mouse, *cxcl12-cxcr4* together with *cxcl12-cxcr7* pathway is one of the most famous in guidance of primordium migration. Previous studies have shown that *cxcl12a, cxcr4b*, *and cxcr7b* are asymmetrically expressed in zebrafish during embryonic development and is crucial for posterior LL cell migration (Aman and Piotrowski 2008; Valentin et al. 2007). We asked whether the expression pattern of chemokines is restricted because of Kdm6b defects. At 48 hpf, *cxcl12a* was found expressed like a narrow stripe along the horizontal myoseptum through in situ hybridization test in control-MO embryos. However, in the *kdm6bb* morphant embryos, the staining of *cxcl12a* was inconsecutive and partially missed (Fig. 4a and 4b). At 32 hpf, *cxcr7b* was strongly recognized in the primordium mainly in the trailing region but absent in the leading zone in control siblings while the expression level of *cxcr7b* was impressively downsized in the *kdm6bb* morphant primordia. In addition, the apparent rosette-shaped staining of *cxcr7b* was completely invisible in *kdm6bb* mutants compared with the controls (Fig. 4c, 4d, and 4g). We further detected a similar expression reduction of *cxcr4b* in the disordered KDM6B morphant primordia. Consistent with the previous data, *cxcr4b* was verified broadly and highly expressed in most regions of the primordium, especially in the leading part in the controls. However, after Kdm6b knockdown, *cxcr4b* expression was dramatically decreased both from the trailing zone to the leading zone (Fig. 4e–4g). To verify our results, we conducted real-time qPCR experiments to examine the mRNA level of *cxcl12a, cxcr4b*, *and cxcr7b*. Consistent with the ISH data, the qPCR analysis revealed significant reduction of relative cxcr7b and cxcr4b level in *kdm6bb*-MO embryos compared to the ConMO group while the change of cxcl12a was not significant between the experimental and control group (Figure S3). Taken together, the WISH data showed that Kdm6b might be a regulator of PLL morphogenesis during primordium migration by modulating chemokine signaling.

### Kdm6b controls the migrating primordium through Wnt and Fgf signaling

**Fig. 5:**
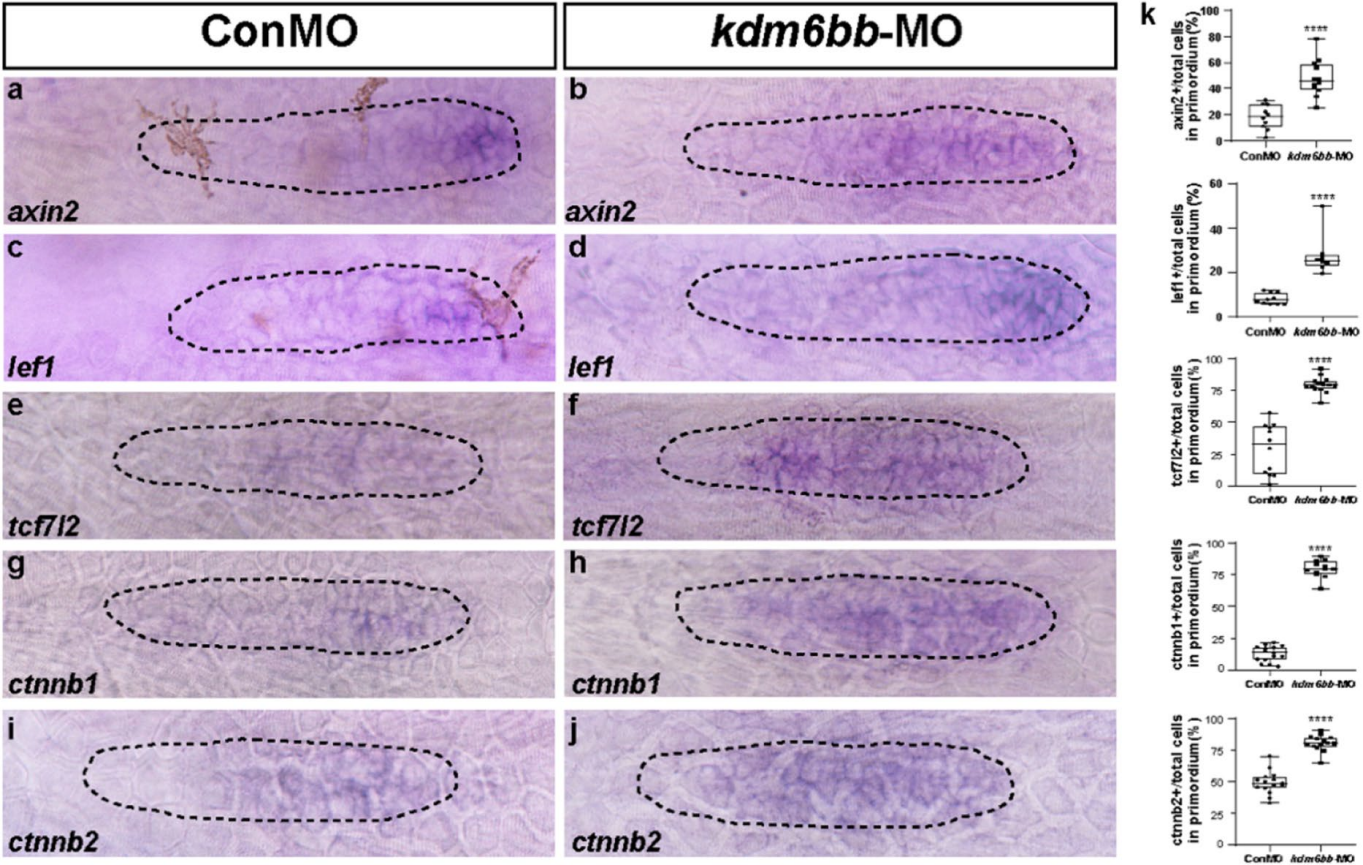
Knockdown of Kdm6b upregulates the Wnt signaling pathway. **a–j** In situ staining of the Wnt signaling target genes, *axin2, lef1, tcf7l2, ctnnb1, and ctnnb2* are all upregulated in *kdm6bb*-MO embryos compared to the controls by in situ hybridization at 32 hpf. **k** The ratio of *axin2, lef1, tcf7l2, ctnnb1, and ctnnb2*-positive cells to total cells in the primordia of controls (ConMO; *n* = 10 embryos for *axin2 and lef1*, respectively; *n* = 12, 15, and 15 embryos for *tcf7l2, ctnnb1, and ctnnb2*, respectively) and experiment group (*kdm6bb*-MO; *n* = 12 embryos for *axin2*, *n* = 8 embryos for *lef1*, *n* = 15 embryos for *tcf7l2*, *n* = 12 embryos for *ctnnb1*, and *n* = 14 embryos for *ctnnb2*).The black dotted lines are drawn to shape the primordium. Data are recorded as mean (minimum and maximum values). ****p < 0.0001

Previous studies concerning the function of canonical Wnt/β-catenin and Fgf signaling have demonstrated that their coordination plays crucial roles in controlling the cell migration and proliferation during early stage of primordia and proneuromast development(Aman and Piotrowski 2008). We therefore asked whether Kdm6b has interactions with the two associated signaling pathways in lateral line primordium migration process. Firstly, we examined the expression level of Wnt target genes *axin2* and *lef1* and found that both genes were restricted in the right and leading edge of the migrating primordium as labeled by dotted lines in control embryos between 32 hpf. In contrast, in *kdm6bb*-MO morphants, expression of both genes, especial the area of *axin2* staining significantly expanded, crossing the leading tip to the trailing region (Fig. 5a, 5b, and 5k), which indicates a negative regulation of *kdm6bb* on Wnt signaling. To confirm our findings, we further detected the expression levels of other Wnt target genes, such as *tcf7l2*, *ctnnb1*, and *ctnnb2*. Our results demonstrated significant upregulation of *tcf7l2*, *ctnnb1*, and *ctnnb2* transcript levels in *kdm6bb*-MO embryos compared with the controls (Fig. 5e–5k).

**Fig. 6:**
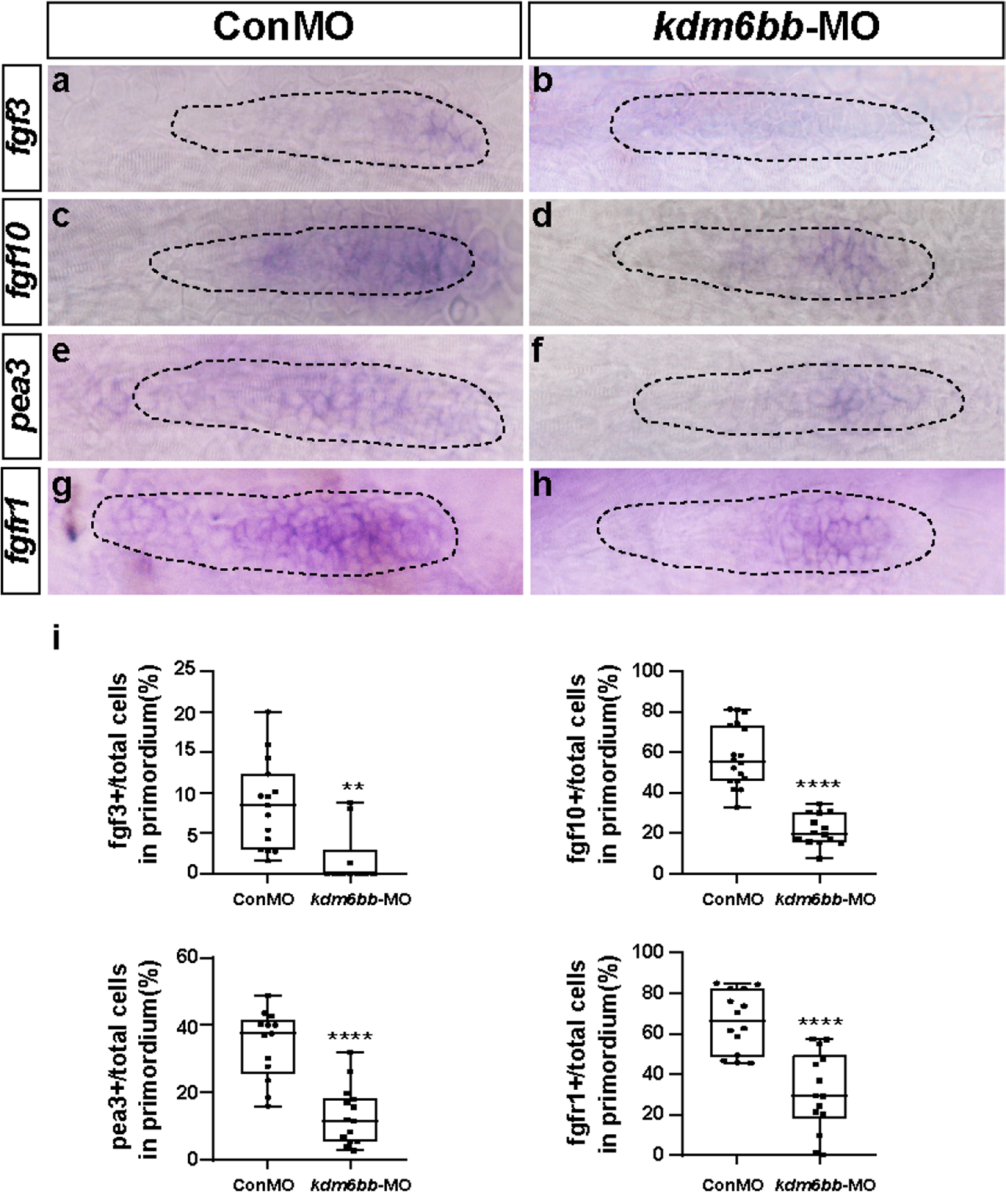
Kdm6b-depletion represses Fgf signaling in zebrafish primordium. **a–h** Corresponding decreased expression of Fgf signaling components including *fgf3*, *fgf10*, *pea3*, and *fgfr1* are presented in *kdm6bb* morphants compared to the control siblings at 32 hpf. **i** The ratio of *fgf3*, *fgf10*, *pea3*, and *fgfr1*-positive cells to total cells in the primordia of controls (ConMO; *n* = 15 embryos for *fgf3*, *n* = 18 embryos for *fgf10*, *n* = 13 embryos for *pea3*, and *n* = 14 embryos for *fgfr1*) and experiment group (*kdm6bb*-MO; *n* = 10 embryos for *fgf3*, *n* = 14 embryos for *fgf10*, *n* = 14 embryos for *pea3*, and *n* = 14 embryos for *fgfr1*).The primordium appearance is shaped by dotted lines. Data are recorded as mean (minimum and maximum values). **p < 0.01, and ****p < 0.0001

The Fgf signaling pathway members *fgf3* and *fgf10* have been described to be the only detectable FGF ligands in the migrating primordia (Nechiporuk and Raible 2008). We next examined the expression level of *fgf3* and *fgf10* in the lateral line system. We collected 30–32 hpf embryos, using WISH staining, we found *fgf3* expression of the controls was restricted in the leading zone, and *fgf10* had a wider expression than *fgf3*. However, lower distributions of *fgf3* and *fgf10* were detected after loss of Kdm6b function in posterior LL primordia (Fig. 6a–6d, and 6i). There was a transcript reduction of *pea3*, a Fgf signaling target downstream gene, in the PLL primordia of the *kdm6bb* variants compared to that of controls (Fig. 6e, 6f, and 6i). Furthermore, the transcript encoding FGF receptor, named *fgfr1*, was found strongly expressed in the control posterior LL. However, in the primordium of *kdm6bb* morphants, *fgfr1* gene expression was inhibited to a minor area (Fig. 6g–6i).

In addition, our real-time quantitative PCR analysis demonstrated the same variation tendency with the ISH semi-quantitative analysis results, especially in the changes of *fgf10*, *pea3*, and *fgfr1* mRNA levels (Figure S3). Collectively, these results indicated that the loss of Kdm6b reduced the expression of Fgf signaling pathway but increased the Wnt/β-catenin signaling in PLL primordia, which might be the reason of disturbed cell migration and proliferation.

## Discussion

The development and morphogenesis of auditory organs involve complicated interplay of multiple genetic and epigenetic factors. Our previous study showed that using GSK-J4 to inhibit H3K27me3 demethylase reduced the proliferative hair cell regeneration in posterior LL neuromasts of zebrafish (Bao et al. 2017). As the specific demethylase of H3K27me3, we hypothesized that Kdm6b would have an impact on the development of zebrafish lateral line. In this study, we investigated the function of Kdm6b during zebrafish posterior LL development. WISH results solidly displayed that Kdm6b was expressed in the migrating primordium and deposited neuromasts of zebrafish in a temporally and spatially controlled manner. Using the antisense MO knockdown technology, we found that the primordium migration was disrupted, the proportion of cell proliferation was severely decreased, and the number of PLL neuromasts in *kdm6bb* morphants was significantly reduced. Moreover, the result of partially restored number of neuromasts in RNA rescue test confirmed the important role of Kdm6b in PLL morphogenesis. Furthermore, the *kdm6bb* variants showed a consistent decreased distribution of *cxcr4b* and *cxcr7b*, and the intact staining of *cxcl12a* was broken off as shown in the in situ hybridization data. In addition, Wnt signaling was upregulated while Fgf signaling was downregulated in the *kdm6bb*-MO embryos. To make our data more convincing, we also conducted the ISH analysis using sense mRNA probe of indicated genes and failed to find any transcript expression, indicating a negative control of sense probe for ISH (Figure S2). Therefore, our research data suggests that Kdm6b might be a novel and hopeful target for hearing loss.

Epigenetic modifications including histone methylation and demethylation and histone acetylation and deacetylation have been found to play dual regulation roles in gene activation or repression, thus active in multiple biological processes by regulating chromatin structure and protein function. For example, the regulation and level of H3K9me2 has been reported to be involved in age-related hearing loss (rat model) (Li et al. 2021), in protection of hair cell from death (mouse damage model) (Yu et al. 2013), and in the process of hair cell regeneration after neomycin damage (zebrafish model) (Tang et al. 2016). The methylated H3K9 and H3K4 are classic examples of antagonistic effects concerning the dynamic modification of histone methylation, which usually the methylated H3K9 is related to negative transcription, but hypermethylation of H3K4 is usually correlated with active gene expression (Briggs et al. 2001; Völkel and Angrand 2007). However, the statement is not absolute. In our previous study, when treatment with the inhibitor of LSD1/KDM1A, the upregulated H3K4me2 level was related to a reduced number of both HCs and SCs in zebrafish larvae (He et al. 2013). In addition, the LSD1 inhibitor 2-PCPA has been confirmed to induce suppression of HC proliferative regeneration after neomycin induced HC damage (He et al. 2016). Similarly, inhibition of H3K9me2 with BIX01294 led to decreased number of HCs during regeneration events following neomycin-induced hair cell loss (Tang et al. 2016). As previously referred, Kdm6b, as an H3K27me3 demethylase, is involved in embryonic development and the formation of the endoderm, mesoderm, and ectoderm (Jiang et al. 2013; Li et al. 2014; Morales Torres et al. 2013; Tang et al. 2014; Zhang et al. 2015). In addition, Kdm6b is also involved in maintaining the pluripotency of mouse embryonic stem cells, assisting the self-renewal and differentiation of cardiovascular cells, and skin repairment (Agger et al. 2009; Apostolou and Hochedlinger 2013; Ohtani et al. 2013; Shaw and Martin 2009), immune system (De Santa et al. 2007; Liu et al. 2015; Salminen et al. 2014; Yan et al. 2014), neurodegenerative disease (Gomez-Sanchez et al. 2013; Tang et al. 2014), and cancer (Anderton et al. 2011; Ramadoss et al. 2012; Wei et al. 2013). The trimethylation of histone H3K27 is related to repressive transcription and gene silence. The reduced H3K27me3 level has been delineated as a biomarker of poor prognosis and invasive tumors, suggesting inhibition of gene expression of H3K27me3 (Bayliss et al. 2016). Loss of H3K27me3 has also been reported to mark aggressiveness of breast cancer, and increased H3K27me3 led to decreased tumor cell growth and migration (Hsieh et al. 2020). Here, our study detected the upregulation of H3K27me3 demethylase by *kdm6bb* knockdown severely disrupted the migration and deposition behavior of zebrafish posterior lateral line, suggesting a similar inactivation role of H3K27me3 in auditory organs compared with cancer. The multiple roles of KDM6B in various organs or systems make it a potential therapeutic target for some diseases; for example, KDM6B is upregulated in blood disorders, and inhibition of its negative regulator AP-1 in *Kdm6b-*deficient hematopoietic stem cells restores the functional defects (Mallaney et al. 2019). In patients with myelodysplastic syndromes and chronic myelomonocytic leukemia, overexpression of KDM6B has been reported. By generating a Vav-*Kdm6b* mouse model, KDM6B is overexpressed in the hematopoietic compartment, resulting in significant hematopoietic defects. However, using the KDM6B inhibitor GSK-J4 can ameliorate the ineffective hematopoiesis observed in Vav-*Kdm6b* mice, suggesting a therapeutic potential for myeloid disorders through targeting KDM6B (Wei et al. 2018). In epithelial ovarian cancer cells, overexpression of KDM6B promoted proliferation, migration, and invasion, while silencing KDM6B inhibited these processes and served as a therapeutic target (Liang et al. 2019). In this study, considering the knockdown strategy by morpholino, we chose co-injection of *kdm6bb* mRNA and morpholino as the complementary strategy, and we found it could partially rescue the defects of PLL.

During migration, the PLL primordial cells are clustered and organized into a radial arrangement called rosettes, which are deposited repeatedly in a stereotyped manner, and then the deposited proneuromasts differentiate into mature neuromasts (Chitnis et al. 2012; Ghysen and Dambly-Chaudiere 2004). The chemokine network involving the broad bindings between ligands and corresponding receptors is well studied for the function of promoting cell migration, particularly in leukocytes, inflammation, heart and vascular system, and lateral line (Hughes and Nibbs 2018). The chemokine ligand-receptor (*cxcl12a-cxcr4b-cxcr7b*) system has been reported to be an important guidance signal of directional migration in zebrafish (David et al. 2002; Li et al. 2004). *Cxcl12a-cxcr4b* signal is mostly restricted in the posterior and leading region of the zebrafish primordium, while *cxcr7b* is also tethered to *cxcl12a* but asymmetrically distributed in the opposite and trailing zone (Aman and Piotrowski 2008). *Cxcl12* is the most elemental ligand that is expressed consecutively along the intermediate segment; however, the in situ staining of *cxcl12a* completely ceased in KDM6B-deficient embryos, resulting in disturbed migration. Similarly, the *cxcl12a* receptors *cxcr7b* and *cxcr4b* were detected in lower expression levels in *kdm6bb* morphants contrary to the controls. This finding uncovers that *kdm6bb* regulates chemokine network to induce the posterior primordium migration of zebrafish.

The Fgf signaling is essential for rosette formation and primordium migration, while the Wnt/β-catenin signal activates the expression of FGF ligands *fgf3* and *fgf10*, and Wnt activation or Fgf reduction both inhibit proper primordium developing patterns (Aman and Piotrowski 2008; Nechiporuk and Raible 2008). To investigate the mechanism of Kdm6b in controlling primordium migration, we performed ISH experiments with probes of the Fgf and Wnt signaling pathways. The results showed that Kdm6b-depletion significantly induced hyper-expression of *axin2* and *lef1*, but hypo-expression of Fgf components (*fgf3*, *fgf10*, *pea3,* and *fgfr1*). The enhancement expression in the region of Wnt signal target genes in KDM6B-deficient primordium might be related to the blocking of Fgf signal in the trailing region.

In conclusion, we found certain functions of Kdm6b in the developing lateral line system, a mechanosensory organ of zebrafish. Kdm6b loss of function led to impaired PLL formation, interrupted migration of primordium, reduced cell proliferation, and depressed deposition of mature neuromasts. We demonstrated that Kdm6b is a decisive element in controlling normal PLL patterns by regulating Fgf and Wnt signaling and *cxcl12a-cxcr7b/cxcr4b* chemokine pathway, but further studies are needed to check the direct or indirect causality within the phenomenon. In addition, previous studies have shown that the H3K27me3 demethylase Kdm6b is associated with inflammation (Burchfield et al. 2015; Kruidenier et al. 2012; Neele et al. 2017; Satoh et al. 2010), so KDM6B may contribute to the treatment and prevention of otitis media related diseases. Our findings provide noteworthy links of epigenetic histone methylation to the development of hearing organ using zebrafish as an experimental model, enrich the theoretical mechanisms of Kdm6b in controlling the cell migration and proliferation behavior, and contribute to a potential therapeutic target of Kdm6b for hearing loss.

## Materials and methods

### Transgenic zebrafish line

All animal experiments of zebrafish were approved by the Institutional Animal Care and Use Committee of Fudan University in Shanghai. The PLL primordia and neuromasts were observed by the *Tg (cldnb: lynGFP)* line (Haas and Gilmour 2006). The embryos were maintained and raised in embryo medium followed standard recipe after spawning in the constant temperature incubator at 28.5 °C (Westerfield 2000). To avoid pigmentation, embryos were incubated with 1-phenyl-2-thiourea (Sigma-Aldrich) from 10 h after birth. The age and stage of embryos during development was recorded as hours post-fertilization (hpf).

### Morpholino injections and mRNA rescue test

*Kdm6bb*-MO, with a sequence of 5′-AAAAGAACATGACTGACCTGGTGTG-3′ and a solution of 5 ng was injected into *Tg (cldnb: lynGFP)* embryos for *kdm6bb* knockdown at one or two cell stage. To avoid side effects by injection handling, control-MO, with a sequence of 5′-CCTCTTACCTCAGTTACAATTTATA-3′ was injected. For mRNA rescue test, 150 pg of *kdm6bb* mRNA generated by the mMESSAGE mMachine Sp6 Kit (Ambion, Austin, TX, US) was co-injected with *kdm6bb* morpholino.

### *Whole-mount *in situ* hybridization*

The whole-mount in situ hybridization (WISH) experiment was manipulated as previously recited (He et al. 2014; Thisse and Thisse 2014). Briefly, after fixation and gradient dehydration, the embryos were warehoused in pure methanol at − 20 °C. Embryos were rehydrated and then digested in 20 μg/ml proteinase K for preparation of hybridization, then embryos were incubated with the probes at 65 °C overnight. After labeling, larvae were rinsed four times in a graded SSC-PBST series and then put in the blocking reagent at room temperature for 1 h. After bonding to the anti-digoxigenin-AP Fab fragment (Roche) overnight at 4 °C, the embryos were washed sufficiently and then incubated with BM purple AP substrate (Roche) for visualized staining in a dark place. The color reaction was discontinued by NTMT and washed thoroughly in PBST. For whole-mount neuromast imaging, specimens were mounted in 100% glycerol, with a Nikon fluorescence stereomicroscope. All image processing was performed with Photoshop and Illustrator software (2018, Adobe).

### Western blot analysis

The extraction of total protein was operated using AllPrep DNA/RNA/Protein Mini Kit (QIAGEN, Hilden, Germany), and the protein concentrations were detected via A BCA protein kit (Thermo Fisher Scientific, Rockford, IL). Then, proteins were separated on SDS–polyacrylamide gels and transferred onto PVDF membranes (Immobilon-P; Millipore, Bedford, MA, USA). The membranes were incubated overnight with rabbit anti-KDM6B/JMJD3 polyclonal antibody (1:500 dilution, Abcam, USA), rabbit anti-Histone H3K27me3 polyclonal antibody (MEMD Millipore Corp, USA), rabbit anti-Histone H3K27me2 polyclonal antibody (MEMD Millipore Corp, USA), or mouse anti-GAPDH monoclonal antibody as reference (1:1000 dilution, Sigma) at 4 °C. The ECL kit (Pierce) was used to visualize the immunoreactive bands and the intensities of the bands were quantified with Fiji (National Institutes of Health).

### BrdU incorporation and analysis

We manually dechorionated the embryos at first, and the embryos were incubated in 15 mM bromodeoxyuridine (BrdU) (Sigma–Aldrich) for 1.5 h. The embryos were collected individually, anesthetized in 0.02% MS-222 (Sigma–Aldrich), and fixed at 4 °C overnight in 4% PFA. After rinsing several times in PBT-2, the control and kdm6bb-deficient embryos were immersed in 2 N HCl for 30 min at 37 °C for blocking of non-specific binding domains. Immunocytochemistry staining was manipulated following the standard procedure after incubation with the primary mouse monoclonal anti-BrdU antibody (1:300 dilution, Santa Cruz Biotechnology) for 1 h at 37 °C and then 4 °C overnight. After washing 3 times in PBT-2, the secondary Cy3 donkey anti-mouse polyclonal antibody (1:300 dilution, Jackson) was added and co-incubated for 1 h at 37 °C successively. The proliferation analysis was conducted by detection of BrdU-positive cells. The fluorescent images were photographed with a multichannel confocal microscope (TCS SP8; Leica), and the further procession of pictures was realized with Photoshop and Illustrator software (2018, Adobe).

#### Real-time PCR quantification

Total RNAs were extracted from the experimental and control embryos at 48 hpf after deletion of yolk sac using TRIzol reagent (Thermo Fisher Scientific) and reverse transcription into cDNA was performed with the Transcriptor First Strand cDNA Synthesis Kit (Roche). To monitor the mRNA levels of desirable genes, quantitative real-time PCR system (LightCycler® 480, Roche) was constructed using SYBR Premix Ex Taq II (Takara Biomedical Technology). Results were analyzed using the ΔΔCt method, and primer sequences were described in Supplementary table 1.

### Statistical analysis

The software GraphPad Prism (version, 8.0c) espoused all statistical analyses. Multiple comparisons were performed using one-way ANOVA, while two-group comparisons were illustrated with a two-tailed Student *t*-test. Statistics were all presented as mean (minimum and maximum values) with all the event plotting labeled, and *p value* < 0.05 was treated as statistically significant.

## Supplementary Information

Below is the link to the electronic supplementary material.

Supplementary file1 (DOCX 1413 KB)

## Data Availability

Not applicable.

## Abbreviations

BrdU: Bromodeoxyuridine
GFP: Green fluorescent protein
HCs: Hair cells
Hpf: Hours post-fertilization
KDMs: Lysine demethylases
KMTs: Lysine methyltransferases
LL: Lateral line
MO: Morpholino antisense oligonucleotide
PLL: Posterior lateral line
SCs: Supporting cells
WISH: Whole-mount in situ hybridization
