# Supplementary material for: The H3K27 demethylase controls the lateral line embryogenesis of zebrafish
Source: Cell Biol Toxicol. 2021 Oct 29;39(3):1137–52. doi: 10.1007/s10565-021-09669-y (PMC10406677; doi:10.1007/s10565-021-09669-y)
Supplement: Supplementary file 1 — Supplementary file1 (DOCX 1413 KB) [file 10565_2021_9669_MOESM1_ESM.docx]

**Supplementary data
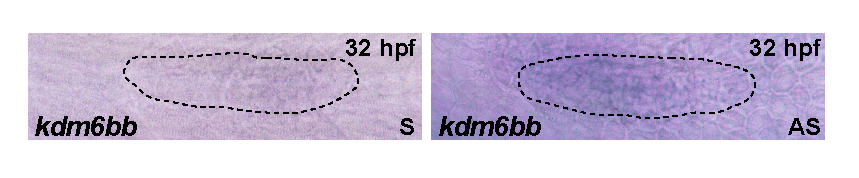
**

**Fig. S1.** The representative S (sense mRNA probe) and AS (antisense mRNA probe) pictures of *kdm6bb* in zebfafish PLL primordia at 32 hpf.


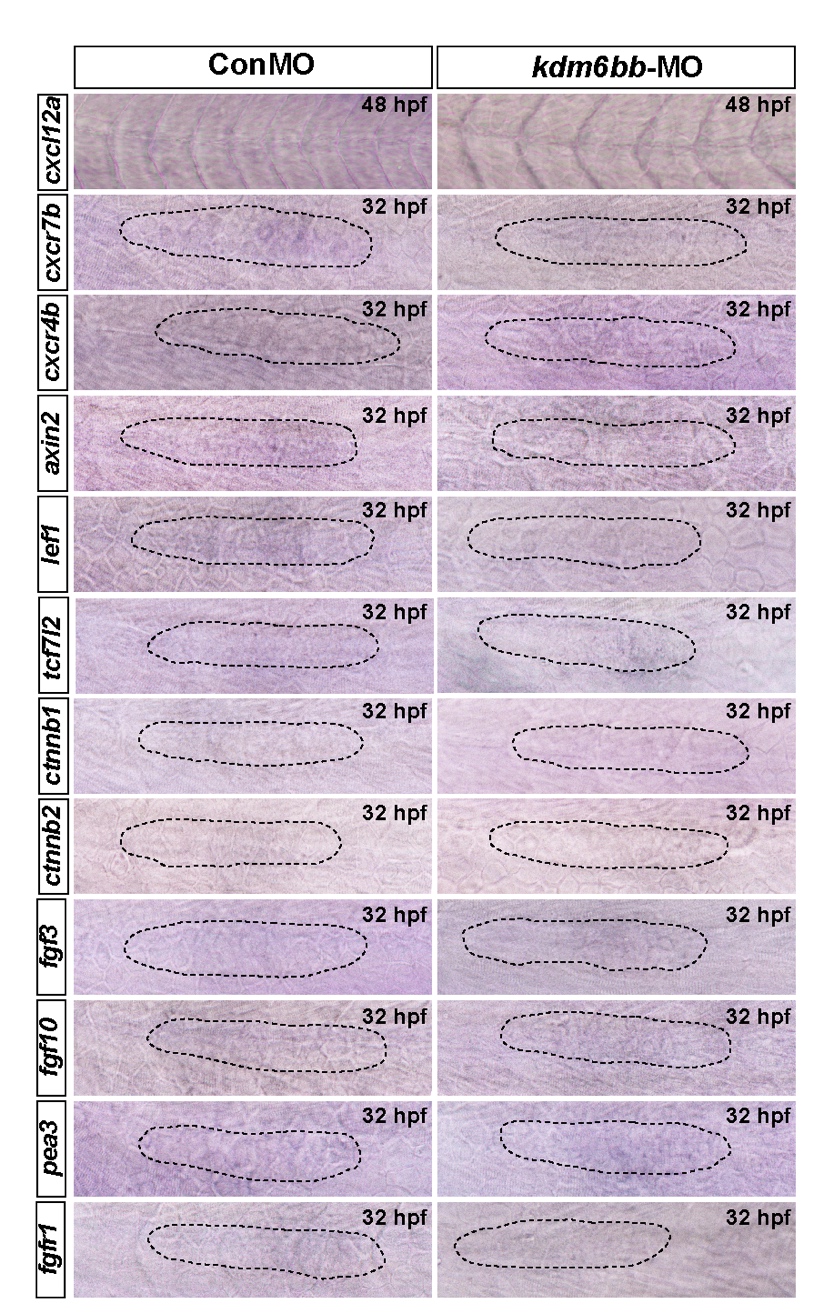


**Fig. S2.** The representative S (sense mRNA probe) pictures of all the chemokines and Wnt and Fgf signaling pathway members used in this study at 32 hpf in zebrafish PLL primordia.


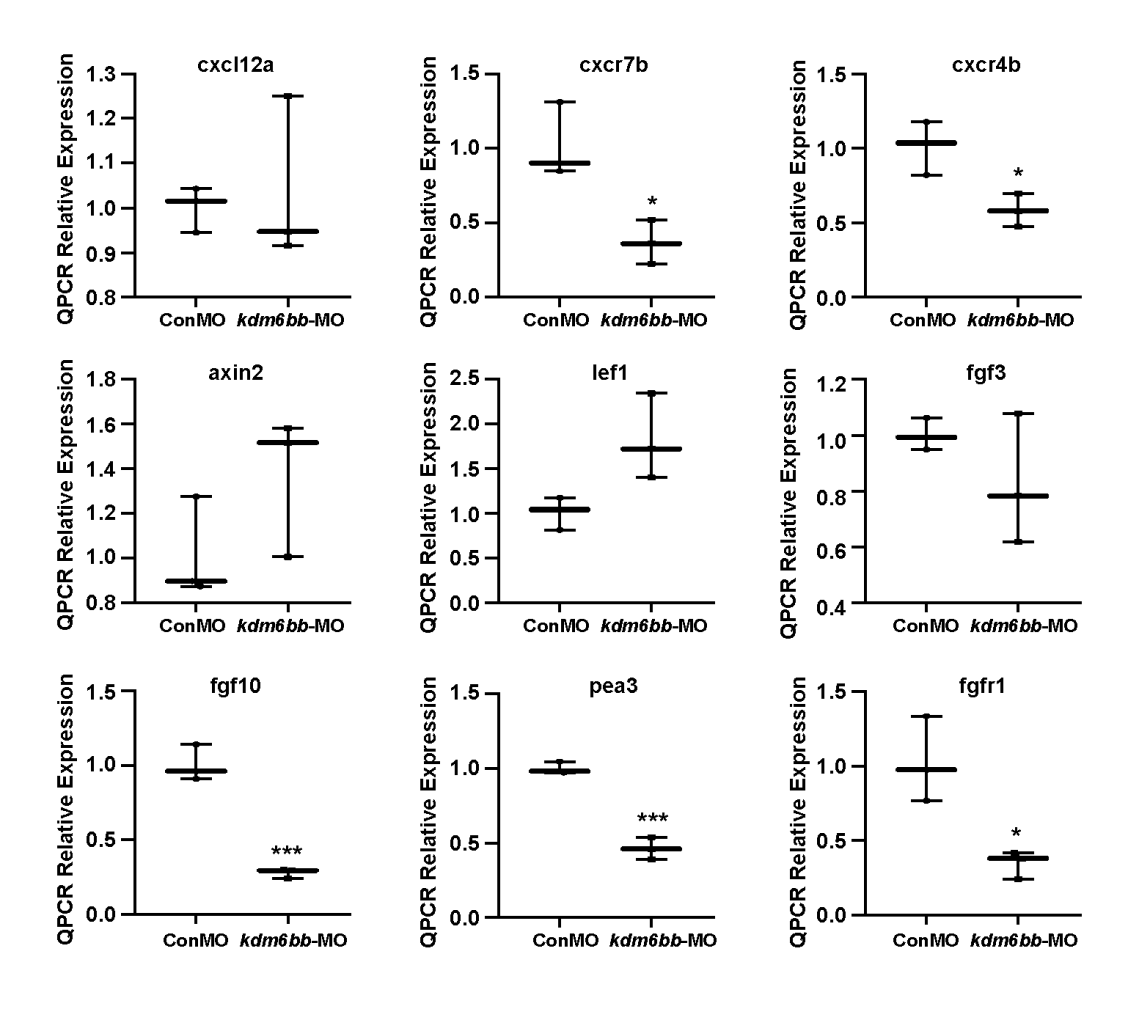


**Fig. S3.** The relative mRNA levels of the indicated genes were normalized to the GAPDH level as determined by RT-qPCR. The results are expressed as the mean (minimum and maximum values) from three independent experiments (n=8 embryos in each group). **p* < 0.05, ****p* < 0.001.

**
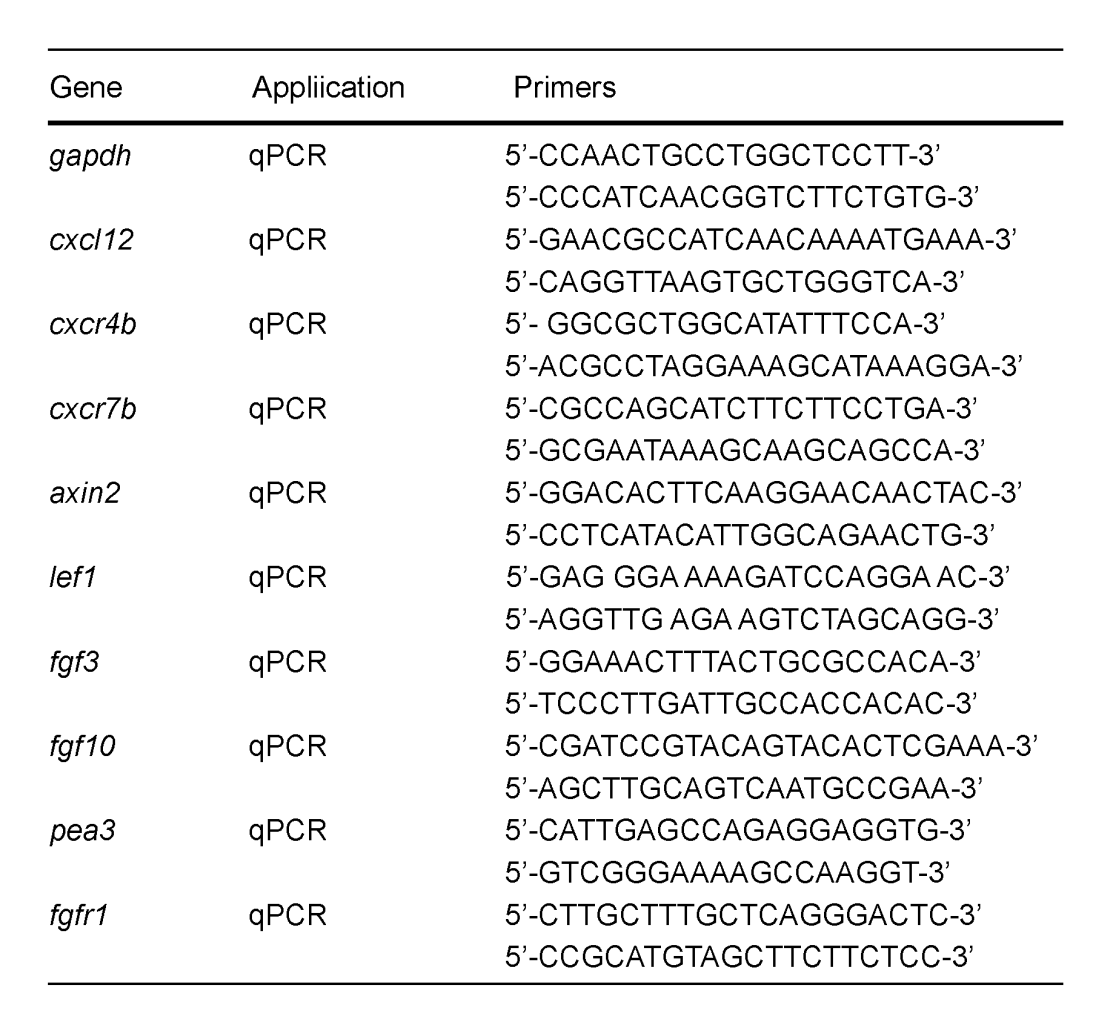
**

**Table. S1.** Primers for Real-Time PCR detection.
